# Modern Lineages of *Mycobacterium tuberculosis* Exhibit Lineage-Specific Patterns of Growth and Cytokine Induction in Human Monocyte-Derived Macrophages

**DOI:** 10.1371/journal.pone.0043170

**Published:** 2012-08-16

**Authors:** Rajesh Sarkar, Laura Lenders, Katalin A. Wilkinson, Robert J. Wilkinson, Mark P. Nicol

**Affiliations:** 1 Division of Medical Microbiology, University of Cape Town, Cape Town, South Africa; 2 National Health Laboratory Services, Groote Schuur Hospital, Cape Town, South Africa; 3 Clinical Infectious Diseases Research Initiative, Institute of Infectious Diseases and Molecular Medicine, University of Cape Town, Cape Town, South Africa; 4 Division of Medicine, Imperial College London, London, United Kingdom; 5 MRC National Institute for Medical Research, Mill Hill, London, United Kingdom; Institut de Pharmacologie et de Biologie Structurale, France

## Abstract

**Background:**

Strains of *Mycobacterium tuberculosis* vary in virulence. Strains that have caused outbreaks in the United States and United Kingdom have been shown to subvert the innate immune response as a potential immune evasion mechanism. There is, however, little information available as to whether these patterns of immune subversion are features of individual strains or characteristic of broad clonal lineages of *M. tuberculosis*.

**Methods:**

Strains from two major modern lineages (lineage 2 [East-Asian] and lineage 4 [Euro-American]) circulating in the Western Cape in South Africa as well as a comparator modern lineage (lineage 3 [CAS/Delhi]) were identified. We assessed two virulence associated characteristics: mycobacterial growth (in liquid broth and monocyte derived macrophages) and early pro-inflammatory cytokine induction.

**Results:**

In liquid culture, Lineage 4 strains grew more rapidly and reached higher plateau levels than other strains (lineage 4 vs. lineage 2 p = 0.0024; lineage 4 vs. lineage 3 p = 0.0005). Lineage 3 strains were characterized by low and early plateau levels, while lineage 2 strains showed an intermediate growth phenotype. In monocyte-derived macrophages, lineage 2 strains grew faster than lineage 3 strains (p<0.01) with lineage 4 strains having an intermediate phenotype. Lineage 2 strains induced the lowest levels of pro-inflammatory TNF and IL-12p40 as compared to other lineages (lineage 2: median TNF 362 pg/ml, IL-12p40 91 pg/ml; lineage 3: median TNF 1818 pg/ml, IL-12p40 123 pg/ml; lineage 4: median TNF 1207 pg/ml, IL-12p40 205 pg/ml;). In contrast, lineage 4 strains induced high levels of IL-12p40 and intermediate level of TNF. Lineage 3 strains induced high levels of TNF and intermediate levels of IL-12p40.

**Conclusions:**

Strains of *M. tuberculosis* from the three major modern strain lineages possess distinct patterns of growth and cytokine induction. Rapid growth and immune subversion may be key characteristics to the success of these strains in different human populations.

## Introduction

*Mycobacterium tuberculosis*, the causative agent of tuberculosis (TB), infects over 2 billion people world-wide and causes 1.7 million deaths annually [1]. The clinical significance of strain variation in *M. tuberculosis* remains controversial, partly since virulence cannot be simply defined in relation to defined virulence characteristics as in other bacterial pathogens, such as *Clostridium tetani* or *Staphylococcus aureus*. Since modern *M. tuberculosis* evolves through single nucleotide substitutions, deletion and duplication events, the population structure is strongly clonal [2]. It is therefore feasible that such clonal lineages may evolve specific virulence characteristics [3].

In a robust phylogenetic study based on genomic deletion analysis, *M. tuberculosis* has been classified into six major lineages. These lineages are highly predominant in specific geographic areas and named according to their geographical distribution: Lineage 1 (also known as Indo-Oceanic lineage), Lineage 2 (also known as East Asian; includes “Beijing”), Lineage 3 (also known as CAS/Delhi), Lineage 4 (also known as Euro-American), Lineage 5 (also known as West African 1) and Lineage 6 (also known West African 2). In the Western Cape region of South Africa the “Lineage 2 (East Asian; includes “Beijing”) and “Lineage 4 (Euro-American; includes LAM3/F11 strains) lineages predominate [4], [5], [6], [7].

Macrophages are the primary site of intracellular replication of *M. tuberculosis*. In humans, large numbers of alveolar macrophages are not readily available for *in vitro* study, therefore matured human monocyte derived macrophages (MDM) derived from peripheral blood mononuclear cells (PBMC) are often used as an *in vitro* model to study *M. tuberculosis* infection in human cells. Activated macrophages in the presence of T cells and inflammatory cytokines are known to be effective in restricting mycobacterial growth.

Lineage 2 strains are transmitted globally with a high tendency to cause outbreaks and multidrug-resistant tuberculosis infection, suggesting that this lineage may possess particular virulence characteristics [8], [9]. Recent reports indicate that lineage 2 strains are emerging in Western Cape region of South Africa [10], [11]. Lineage 2 strains replicate to high bacillary loads in lungs of infected mice [12]. Strains from this genotype have been shown to replicate more rapidly in the human cell culture model [13], [14], [15]. Several human and animal studies have suggested that the propensity of lineage 2 strains to induce lower levels of protective Th1 cytokines (such as TNF, IL-12 and IFN-γ) may be, in part, responsible for enhanced virulence [16], [17], [18], [19], [20].

Lineage 3 is related to lineage 2 by genome-based phylogeny [7] but is more restricted in geographical location and largely confined to the Indian subcontinent and some regions in East Africa that have experienced a significant Indian migration. This lineage is also common in the United Kingdom amongst Indian populations [21] but is uncommon in Cape Town. Two studies have reported that CAS (central Asian) strains, which belong to lineage 3, grow slowly in liquid broth [19], [22]. Furthermore, a lineage 3 strain (CH), responsible for an outbreak in the United Kingdom, induced less protective IL12p40 and more anti-inflammatory IL-10 than the reference strain H37Rv [22].

Lineage 4 is distributed throughout Europe, America, parts of Africa and the Middle East. This lineage includes Haarlem, Latin American Mediterranean (LAM), X and T families of strains [4], [23]. Strains belonging to the LAM3 family, characterised by the deletion RD761 (RD: region of differentiation) are common in South Africa [2]. The reference strain H37Rv also belongs to lineage 4. H37Rv grows well in the monocyte derived macrophage model [24] and induces similar patterns of cytokines as Harlem and LAM strains in human macrophages [20].

The above experimental evidence suggests that lineages of *M. tuberculosis* may be characterised by lineage-specific patterns of growth and cytokine induction. The aim of this study was to determine whether strains of *M. tuberculosis* from the two major circulating lineages in South Africa (lineage 2 and lineage 4) demonstrate such lineage-specific characteristics *in vitro* models, and whether such patterns are conserved amongst several representatives of these lineages. We also wanted to compare these patterns with those associated with representatives of the lineage 3. We therefore studied *in vitro* growth rate in liquid broth, intracellular growth rate in monocyte derived macrophages and early pro-inflammatory cytokine induction by strains from each of these different lineages.

## Materials and Methods

### Ethics statement

The study was conducted with ethical approval from the Faculty of Health Sciences research ethics committee, University of Cape Town (Reference 261/2008).

### Selection of *M. tuberculosis* strains

We selected strains of *M. tuberculosis* from a previously described collection of isolates from children from the broader Cape Town region of South Africa [6]. Strains from children with tuberculosis are likely to be representative of circulating *M. tuberculosis* strains in the region, as childhood tuberculosis classically follows exposure to an infectious adult source case. We performed spoligotyping and multiple interspersed repetitive unit-variable number tandem repeat (MIRU-VNTR) analysis in order to identify the major strain lineages and strain clusters (Table 1). We selected strains from the major clusters within each of the most common lineages in this strain collection, including three lineage 2 (Beijing) strains (two from the RD181, RD150 sublineage and one from the RD181 sublineage) [25] and three lineage 4 (LAM3/F11) strains. In addition, we selected two lineage 3 (CAS) strains, the previously described CH strain as well as a second lineage 3 strain from the Cape Town paediatric collection. H37Rv was used in all assays as a reference strain.

**Table 1:** Genotype of selected *M. tuberculosis* strains.

Strain	MIRU-VNTR	Spoligotype	Genotype
H37Rv	243132253233552	777777477760771	Lineage 4, Laboratory strain
RXH248	442335464485372	000000000003771	Lineage 2, Beijing
RXH24	442345553575482	000000000003771	Lineage 2, Beijing
RXH360	442325554475472	000000000003771	Lineage 2, Beijing
CH	522355424275384	703777740003731	Lineage 3, CAS2
RXH379	4423664?? 285373	702777740003771	Lineage 3, CAS1
RXH266	442344442253172	774177407760771	Lineage 4, LAM3
RXH202	442344442253172	776177607760740	Lineage 4, LAM3
RXH6	442365542253173	774377007760771	Lineage 4, LAM3

### Preparation of mycobacterial culture

To ensure a synchronous growth phase amongst all strains, *M. tuberculosis* was grown at 37°C in a shaking incubator (120 rpm) to mid log phase (0.6 to 0.9 OD) in Middlebrook 7H9 broth (Difco, Detroit, MI, USA) containing 0.2% glycerol, 0.05% Tween 80, and 10% albumin-dextrose-catalase (ADC) growth enrichment (Becton Dickinson, Cockeysville, MD, USA). Multiple vials were stored at −80°C until further use. A new vial of bacilli was thawed before each experimental procedure. The colony forming unit (CFU) concentration of stock vials was calculated by serial dilution and plating in multiple replicates on Middlebrook 7H11 agar (Difco, Detroit, MI, USA) containing 0.5% glycerol and 10% oleic acid-albumin-dextrose-catalase (OADC) growth enrichment (Becton Dickinson, Cockysville, MD, USA).

### Axenic growth assays *M. tuberculosis* growth in liquid 7H9 broth

For growth in axenic media, frozen stock of known CFU concentration were freshly thawed and multiple replicates of 10^5^ CFU/ml of bacilli of *M. tuberculosis* were inoculated into pre-warmed 7H9 Middlebrook broth supplemented with .2% of glycerol, .05% Tween 80, and 10% ADC growth enrichment and cultured in a shaking incubator (120 rpm) at 37°C for 360 h. To determine growth rate, aliquots were withdrawn at intervals and plated for CFU enumeration by serial dilution and plating in multiple replicates onto 7H11 agar containing 10% OADC.

### Monocyte isolation, culture and maturation to monocyte derived macrophages (MDM)

MDM were prepared from healthy donor buffy coats supplied by South African Blood Transfusion Services. Peripheral blood mononuclear cells (PBMC) were separated by centrifugation over Ficol-paque Plus (Pharmacia, Uppsala, Sweden). Cells were washed in RPMI, pooled and counted. Cells were incubated in large tissue culture flask (175 cm^2^) at 300×10^6^ PBMC per 25 ml RPMI per flask for 2 hours at 37°C in 5% CO_2_. This step allows monocytes to adhere to the flask. Non-adherent cells were removed by three washes with 10 ml of pre-warmed RPMI medium (Sigma-Aldrich, Ayrshire, UK). Finally, 10 ml of ice-cold phosphate buffered saline (PBS) was added and the flask incubated at 4°C for 20 minutes. Using a long handled scraper, monocytes were dislodged from the bottom of the flask and pooled in a 50 ml falcon tube for counting. Cells were plated in R10 medium (RPMI containing 10% foetal calf serum (FCS)) at a concentration of 2×10^5^ cells/well in a 96 well tissue culture plate and cultured at 37°C, in 5% CO_2_, for 6 days in R10 medium to mature into macrophages.

### MDM infection and intracellular growth assay

For the intracellular growth assay, frozen *M. tuberculosis* stock were freshly thawed and reconstituted at room temperature at the time of infection. Before infection, each stock vial was plated for CFU enumeration to reconfirm the standard concentration of the inoculum. Adherent MDM were co-cultured in duplicate with bacilli at 1∶1 ratio in R10 medium (RPMI+ non heat-inactivated 10% FCS). After 4 h of incubation, extracellular bacteria were removed gently by washing four times with pre-warmed PBS. After 4, 24, 48 or 96 hours infected MDM were subjected to complete lysis by mixing with 100 µl of 0.1% SDS and incubated at room temperature for 12 mins. Lysates were mixed thoroughly for ten times, serially diluted, and plated, in triplicate, on 7H11 agar plates. Plates were incubated for 3–4 weeks at 37°C and CFU enumerated.

### Cytokine Analysis

Culture supernatants from control and infected MDM cells were harvested after 48 h and frozen at −80°C. Sterile-filtered culture supernatants were assayed using an enzyme-linked immunosorbent assay (ELISA) kit according to manufacturer's instructions (BD Biosciences Pharmingen, San Diego, CA, USA) to measures levels of tumor necrosis factor (TNF) and IL12p40.

### Statistical analysis

Graphpad Prism 5.00 was used for statistical analysis of intracellular growth in monocyte derived macrophages and cytokine assay. The intracellular growth index in MDM infection was compared amongst strains from different groups using the unpaired t test at each time point. Cytokines were analysed using the non-paramatric Mann Whitney test. Axenic growth curve analysis was performed with one-way ANOVA and Bonferroni's post hoc test.

## Results

### Mycobacterial growth in broth

**Figure 1:**
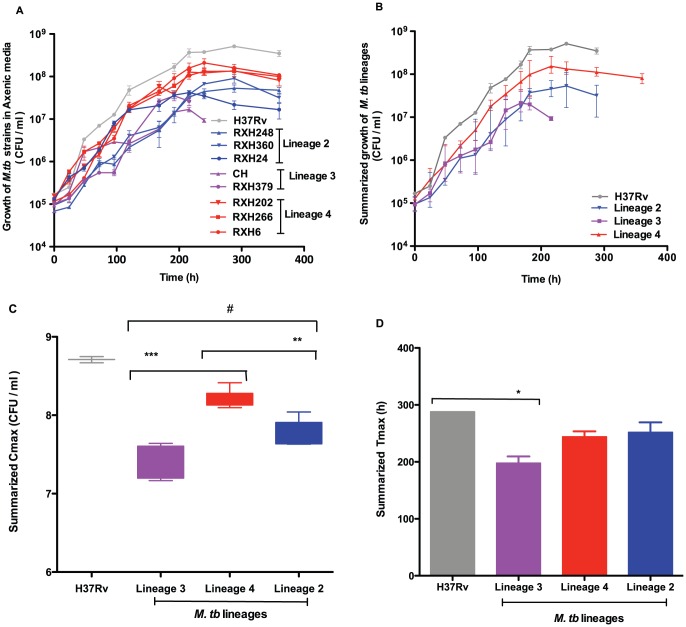
Growth of *M. tuberculosis* strains in liquid 7H9 broth. Fig. 1 (A) Growth of individual *M. tuberculosis* strains from different lineages in liquid 7H9 broth. Data represent the mean and range (n = 2) values. Fig. 1 (B) Summarized growth of *M. tuberculosis* strain lineages. Fig. 1 (C) Comparison of C_max_ (maximum point on the growth curve) for different strain lineages. Lineage 4 strains showed higher C_max_ than lineage 2**(p = 0.01) and lineage 3*** strains (p = 0.001). H37Rv showed significantly higher C_max_ than all clinical strains^#^ (p = 0.001) Fig. 1 (D) Comparison of T_max_ (time to reach to C_max_) for different strain lineages. H37Rv reached T_max_ significantly faster than lineage 3 strains* (p = 0.05).

*M. tuberculosis* strains belonging to different *M. tuberculosis* lineages were grown in liquid 7H9 Middlebrook broth to evaluate the growth rate amongst different lineages in axenic media (two independent cultures, each performed in triplicate) (Fig. 1A and 1B). We used C_max_ and T_max_ to compare growth of various strains. C_max_ is defined as the peak point on the bacterial growth curve (maximum number of colony forming units) whilst T_max_ is the time required to reach C_max_. H37Rv grew to a higher C_max_ than clinical strains (Table 2). Lineage 4 strains showed a higher C_max_ than clinical strains from other lineages. (Fig. 1C) lineage 2 strains showed an intermediate level of growth whilst lineage 3 strains had the lowest C_max_ (C_max_, H37Rv vs lineage 4/lineage 2/lineage 3 all p<0.0001; lineage 4 vs lineage 2 p = 0.0024; lineage 4 vs lineage 3 p = 0.0005). T_max_ for H37Rv was 288 h. Lineage 3 strains showed significantly shorter T_max_ than H37Rv (p<0.05) (Fig. 1D).

**Table 2:** Strain wise comparison of M. tuberculosis growth in axenic media based on Cmax # and Tmax # # values.

Strain	Lineage	C_max_ (CFU/ml)	T_max_ (h)
H37Rv	Laboratory strain	5.14±0.68×10^8^	288
RXH24	Lineage 2	4.50±0.02×10^8^	204
RXH248	Lineage 2	5.33±1.4×10^7^	288
RXH360	Lineage 2	9.07±2.7×10^7^	264
CH	Lineage 3	1.69±0.34×10^7^	216
RXH379	Lineage 3	3.68±0.97×10^7^	180
RXH202	Lineage 4	1.44±0.082×10^8^	240
RXH266	Lineage 4	1.46±0.29×10^8^	252
RXH6	Lineage 4	2.10±0.70×10^8^	240

# C_max_ : maximum number of colony forming units,

# # T_max_ : Time required to reach C_max_. Data represent mean ± SD obtained from two separate growth experiments.

### Mycobacterial growth in the monocyte derived macrophage model

**Figure 2:**
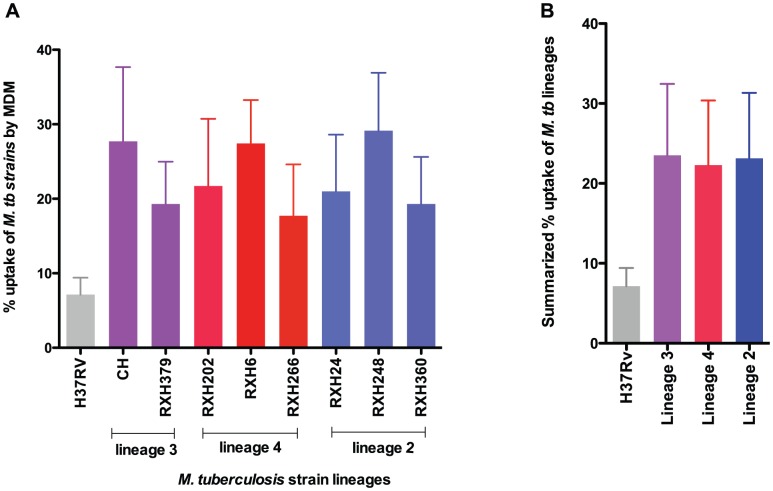
Strain uptake by monocyte derived macrophages. Fig. 2 (A) Uptake of *M. tuberculosis* strains by MDM (4 h CFU calculated as a percentage of initial inoculum). Fig. 2 (**B**) Summarized uptake of *M. tuberculosis* strain lineages by MDM. H37Rv showed significantly lower uptake by the than clinical strains^#^ (p<0.001). Data represent the mean ± SD (n = 7).

We used the MDM infection model to evaluate differences in intracellular growth in different donors (n = 7). We determined initial strain uptake (after 4 h) by expressing the number of CFU at the 4 hour time point from MDM lysates as a percentage of the initial inoculum (Fig. 2). H37Rv showed lower strain uptake by MDM than the clinical strains tested (p<0.001). There were no significant differences in strain uptake between clinical strains.

**Figure 3:**
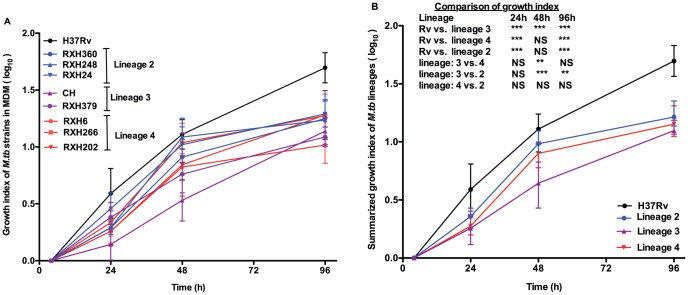
Growth index reflecting the intracellular growth of *M. tuberculosis* strains in MDM. Fig. (3A) Growth index of *M. tuberculosis* strains (calculated by the CFU at each time point divided by the CFU at initial time point). Fig. (3B) Summarized growth index of *M. tuberculosis* strain lineages. Data represent the mean ± SD (n = 7). Statistical significance is denoted as *p<0.05, **p<0.01, ***p<0.001.

To compare intracellular growth amongst different strain lineages, we determined a growth index, calculated from the log_10_ of number of CFU at each time point divided by the log_10_ of number of CFU at the 4 h time point. Growth of all clinical strains was slow for the first 24 hours. Lineage 2 and lineage 4 strains grew faster over the first 48 hours than lineage 3 strains (lineage 3 vs. lineage 4 : p<0.01, lineage 3 vs. lineage 4: p<0.001). lineage 2 strains grew significantly faster than lineage 3 strains (p<0.01) over 96 hours (the mean growth index of lineage 4, lineage 2, lineage 3 and H37Rv was 1.19±.22, 1.25±.17, 1.11±.10 and 1.69±.13 respectively over 96 hours) (Fig. 3). Despite low initial uptake, H37Rv multiplied more rapidly in MDM than clinical strains (H37Rv vs. lineage 2 and lineage 4: p<0.001 at 24 h and 96 h, H37Rv vs. lineage 3 : p<0.001 at all time points). As an alternative approach to compare the growth rate amongst these strains, we have also determined the doubling time of these strains (Doubling time was calculated based on the mean numbers of CFU at 4 hours and 96 hour) (Table 3). The mean doubling time of lineage 2 strains was 23.34±3.0 h, lineage 4 strains 25.10±4.67 h, lineage 3 strains 26.14±2.45 h and H37Rv 17.12±1.61 h (mean ± SD) over the 96 hour time period.

**Table 3:** Doubling time+ for *M. tuberculosis* strains from different lineages in human monocyte derived macrophages.

Strain	Lineage	24 hrs	48 hrs	96 hrs
H37Rv	Lab strain	13.71±2.17	13.16±1.44	17.12±1.61
RXH24	Lineage 2	17.23±2.59	14.41±1.59	22.85±2.11
RXH360	Lineage 2	38.99±13.2	16.31±1.82	23.47±2.11
RXH248	Lineage 2	25.78±2.71	13.52±1.53	23.72±2.10
CH	Lineage 3	44.97±8.9	29.79±3.75	25.60±2.09
RXH379	Lineage 3	20.93±3.05	19.72±2.18	26.69±2.08
RXH202	Lineage 4	35.14±7.45	18.92±2.8	23.14±2.26
RXH266	Lineage 4	24.48±4.08	14.21±1.57	23.03±2.05
RXH6	Lineage 4	36.37±9.91	17.66±1.81	29.03±2.59

+ Doubling times = t/3.3 log (b/B) (t = time, B = initial colonies, b = final colonies). Data represent means ± S.D from n = 7 donors.

### Cytokine induction

**Figure 4:**
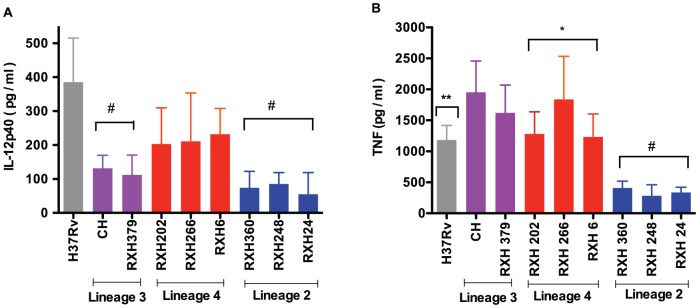
Cytokines detected in culture-supernatant (at 48 h) from MDM infected by different strains of *M. tuberculosis*. Fig. 4 (A) IL-12p40 (n = 6 donors). H37Rv and lineage 4 strains induced significantly higher level of IL-12p40 as compared to lineage 3^#^ and lineage 2^#^ lineages (p<0.001 for both comparisons) Fig. 4 (B) TNF (n = 8 donors). Lineage 3 strains induced significantly higher levels of TNF as compared to lineage 2^#^ (p = 0.001), lineage 4* (p<0.05) and H37Rv** (p = 0.01). H37Rv and lineage 4 strains induced higher level of TNF than lineage 2 strain (lineage 4 vs lineage 2 p = 0.001, H37Rv vs lineage 2 p = 0.01). Data represent the median and range.

We investigated the release of TNF (n = 8 donors) and IL-12p40 (n = 6 donors) from monocyte-derived macrophages (MDM) during 48 hours of infection with *M. tuberculosis* strains of various lineages at 48 hours (Fig. 4). Lineage 2 and lineage 3 strains induced lower levels of IL-12p40 when compared to H37Rv (H37Rv: 385±39.89 pg/ml, lineage 2: 91±17.85 89 pg/ml, lineage 3: 123.8±10.1589 pg/ml, median ± S.E, p<0.001) and lineage 4 strains (lineage 4: 205±14.84 pg/ml, median ± S.E, p<0.001) (Fig. 4A). Further, the release of TNF by MDM infected with lineage 2 strains was significantly reduced when compared to H37Rv (H37Rv: 1253±83.52 pg/ml, lineage 2: 362±27.58 pg/ml, median ± S.E, p<0.01) and other lineages (lineage 3: 1818±123 pg/ml, lineage 4: 1207±112 pg/ml, median ± S.E, p<0.001). In contrast to IL-12p40, higher levels of TNF were induced by lineage 3 strains when compared to H37Rv (p<0.01) and lineage 4 strains (p<0.05). Induction of both TNF and IL-12p40 by lineage 4 strains was similar to that induced by H37Rv.

## Discussion

In this study, we compared lineage-specific *in vitro* phenotypes of the two prevalent strain lineages from the Western Cape region of South Africa with those of the laboratory strain H37Rv as well as strains from lineage 3. We assessed two virulence-associated characteristics in our study: mycobacterial growth (in liquid broth and MDM) and proinflammatory cytokine induction in MDM. We clearly demonstrated lineage-specific patterns of growth and cytokine induction.

The laboratory-reference strain H37Rv grew most rapidly in liquid culture, suggesting that H37Rv is highly adapted to laboratory media. Interestingly, there were clear lineage-specific differences in growth patterns in axenic media, with lineage 4 strains reaching a higher C_max_ than the other two lineages and lineage 3 strains peaking early at a lower C_max_. Lineage 2 strains had an intermediate phenotype. Our results are in line with previous findings which showed slow growth of lineage 3 (CAS strain) in 7H9 broth [19], [22].

Several previous studies have assessed intracellular growth of *M. tuberculosis* in human macrophages as a marker of virulence [13], [15], [26], [27]. Individual strains from lineage 2 (Beijing) have been shown to grow more rapidly than comparator strains in *in vitro* human cell culture models using MDM or monocyte or human macophage cell lines [13], [14], [15]. Zhang *et al* utilized the monocyte derived macrophage model to study the correlation between the extent of the spread of *M. tuberculosis* strains in a Los Angeles community setting and the ability of the strains to grow in human macrophages. Multiple isolates of *M. tuberculosis* strain 210 (a Beijing-family strain [lineage 2] responsible for an outbreak in Los Angeles), grew more rapidly than small cluster or unique cluster strains in human MDM [14]. In contrast Shon *et al* found no significant difference in growth of a hypervirulent Beijing strain (K-strain) [lineage 2] from Korea compared with H37Rv in murine bone marrow derived macrophages [18].

We found that lineage 2 and lineage 4 strains grew more rapidly than lineage 3 strains during the first 48 hours but that growth declined over the next 48 h. In contrast, H37Rv continued to grow more rapidly than all clinical strains. Initial uptake by MDM was lowest for H37Rv, but similar for all clinical strains. There is evidence of variation in phagocytosis by different strains [28]. For example Schlesinger *et al* found that phagocytosis of two virulent strains (H37Rv and Erdman) by human monocyte derived macrophages is mediated by the mannose receptor in addition to complement receptor (CR1, CR3 and CR4) whereas the avirulent strain H37Ra uses only complement receptor for phagocytosis [29]. It is unclear whether the differential uptake which we demonstrated is due to differential expression of such pathogen-associated molecular patterns.

There are a number of limitations of this *in vitro* model. Short term growth in macrophages only partially reflects the more lengthy and complex pathogenesis in humans, we were unable to distinguish bacterial uptake from simple adherence to macrophages and PBMC-derived macrophages differ phenotypically from alveolar macrophages [30]. Rapid growth in macrophages may not necessarily represent a virulence characteristic [18]. Furthermore, due to the limited number of MDM available, we were only able to compare a few strains from each lineage. All of these strains (except CH) were isolated from South African patients, and only represent specific sub-lineages. They do not therefore represent the global diversity of ‘modern’ TB strains.

Early proinflamatory cytokine secretion is a hallmark of *M. tuberculosis* infection. IL-12 production is essential to induce a protective Th1 response [31]. In humans, mutations in the IL-12p40 and the IL-12 receptor genes result in enhanced susceptibility to mycobacterial infection [32], [33]. TNF is produced by monocytes and macrophages in early infection and plays a key role in protective immunity during tuberculosis infection. In humans, neutralization of TNF using anti-TNF drugs increases the risk of reactivation of latent tuberculosis [34], [35], [36]. Hirsch *et al*. showed that TNF inhibits the growth of *M. tuberculosis* within alveolar macrophages [37].

Several studies have reported that heterogeneity exists in the cytokine response induced by various genotype of *M. tuberculosis* [20], [22]. Modern lineages (lineage 2, lineage 3 and lineage 4) were shown to induce lower levels of proinflamatory cytokines when compared with ancient lineages (lineage 1, lineage 5 and lineage 6) [38]. Lineage 4, a modern *M. tuberculosis* lineage, consists of a diverse group of *M. tuberculosis* strains. Haarlem and LAM strains belonging to this lineage induced similar level of TNF, IL-6, IL-10 and GRO-α as H37Rv in human macrophages [20]. Another outbreak strain, CDC1551, which is a representative of lineage 4 (belonging to the X family) induces high levels of pro-inflammatory cytokines in monocyte culture [39], [40]. We studied only modern lineages of *M. tuberculosis* and were able to demonstrate distinct patterns of cytokine induction by representatives of the three major modern strain lineages. Lineage 4 (LAM3) strains induced similar levels of cytokine to the reference strain H37Rv. Lineage 3 strains have been reported to either induce similar levels of proinflammatory cytokines to lineage 2 strains (low levels) or to induce similar levels to H37Rv (higher levels) [19], [20], [22]. Our findings are in line with those of Newton and colleagues [22] who described high TNF and low IL-12p40 secretion by MDM infected by a lineage 3 strain.

A recent study has demonstrated that ancient Beijing strains (lineage 2) from Brazil (characterized by the absence of an insertion sequence 6110 within the noise transfer function region) induced similar levels of TNF and IL-10 to H37Rv whilst modern MDR Beijing strains (lineage 2) isolated in Russia, induced low TNF and high IL-10 in the THP1 macrophage cell line [41]. In contrast, another study has found that Beijing strains (lineage 2), irrespective of subfamily, showed an immune phenotype of low level of TNF, IL-6, IL-10 and GRO-α [20] production as compared to H37Rv and other genotypes of *M. tuberculosis* in human macrophages. Low proinflamatory cytokine induction by lineage 2 (Beijing strains) has been repeatedly reported in various human cell culture models including human monocyte-derived macrophages and the THP1 cell line [14], [17], [18], [19], [20], [41]. Our results, with selected modern Beijing strains from South Africa are consistent with other studies showing low levels of proinflamatory cytokine secretion by infected human MDM.

In summary, we have demonstrated that different modern *M. tuberculosis* lineages exhibit lineage-specific patterns of IL12p40 and TNF induction, with lineage 4 strains inducing high levels of both cytokines, lineage 2 strains low levels of both cytokines and lineage 3 strains high levels of TNF but low levels of IL12p40. This study suggests that modern strain lineages of *M. tuberculosis* may possess lineage-specific patterns of growth and cytokine induction. These in vitro characteristics may reflect different strategies that *M. tuberculosis* employs to exploit ecological niches in different human populations.
